# Genomic analysis of early ST32 *Acinetobacter baumannii* strains recovered in US military treatment facilities reveals distinct lineages and links to the origins of the Tn*6168 ampC* transposon

**DOI:** 10.1093/jac/dkae454

**Published:** 2024-12-16

**Authors:** Liam A Tobin, Eradah Abu Sabah, Francois Lebreton, Garry S A Myers, Patrick T McGann, Mehrad Hamidian

**Affiliations:** Australian Institute for Microbiology and Infection, University of Technology Sydney, Ultimo, NSW 2007, Australia; Australian Institute for Microbiology and Infection, University of Technology Sydney, Ultimo, NSW 2007, Australia; Multidrug Resistant Organism Repository and Surveillance Network, Walter Reed Army Institute of Research, Silver Spring, MD, USA; Australian Institute for Microbiology and Infection, University of Technology Sydney, Ultimo, NSW 2007, Australia; Multidrug Resistant Organism Repository and Surveillance Network, Walter Reed Army Institute of Research, Silver Spring, MD, USA; Australian Institute for Microbiology and Infection, University of Technology Sydney, Ultimo, NSW 2007, Australia

## Abstract

**Objectives:**

To study the population structure and genomic characteristics, including antimicrobial resistance genes, plasmid types and surface polysaccharide type, of the globally distributed *Acinetobacter baumannii* belonging to ST32 (Institut Pasteur scheme).

**Methods:**

Antibiotic resistance phenotype for 19 antibiotics was determined using Vitek 2. Whole-genome sequencing was performed using the Illumina MiSeq platform. Genomes were assembled using *Newbler*. Phylogenetic analysis was done by determining the core-genome alignments using *Panaroo* v1.3, analysed in IQ-Tree2 v2.2.0.3 to construct Maximum Likelihood trees using the *RaxML* software. Resistance genes and IS were identified using the *Abricate* programme, and *ISFinder* databases.

**Results:**

One hundred and thirty-three (*n *= 133) ST32 *A. baumannii* isolates were analysed in this study. These genomes originated mainly from US military treatment facilities (*n *= 113), but also included additional publicly available genomes in GenBank (*n *= 20) recovered from a broad geographic distribution extending to Asia and South America. Phylogenetic analysis of all 133 genomes revealed at least four clades, with over 80 genomes forming a tightly clustered branch, suggesting they are likely to represent outbreak strains. Analysis of the *ampC* region showed that ST32 strains played a significant role in the formation of the widely distributed *ampC* transposon, Tn*6168*, and supplying DNA segments containing an ISAba1-*ampC* from ST32s via homologous recombination.

**Conclusions:**

ST32 strains played a significant role in the evolution of antibiotic resistance in several widely distributed sequence types including ST1 (global clone 1) and ST3.

## Introduction

Bacterial antibiotic resistance is a significant public health threat and a growing global concern.^1^  *Acinetobacter baumannii* is a particularly concerning pathogen because of its resistance to last resort antibiotics such as carbapenems.^2^ Like other Gram-negative bacteria, the primary mechanism of resistance in *A. baumannii* is the acquisition of antibiotic resistance genes via mobile genetic elements (MGEs), including genomic islands (GIs), plasmids and transposons.^1–4^ However, in addition to MGEs, resistance gene acquisition events can also occur via other mechanisms. For instance, resistance to third-generation cephalosporins often results from the insertion of an ISAba1 (or ISAba125) element upstream of the chromosomal *ampC* gene, introducing a strong promoter that significantly enhances expression. This leads to high-level resistance to third-generation cephalosporins and beta-lactam inhibitors like sulbactam. IS acquisition can arise either through the classic ISAba1 insertion or via homologous recombination, where an exogenous DNA segment containing an ISAba1-activated *ampC* gene integrates into the chromosome.^1^ Together with the acquisition of MGEs, clonal expansion has also significantly contributed to the global spread of resistant clones of *A. baumannii*.^1^ Most globally distributed strains belong to a limited number of major sequence types (STs) such as ST1, ST2, ST10, ST15, ST25, ST79 and ST85 with ST2 being by far the most encountered ST.^1,5^ However, except for ST1 and ST2, which have been extensively studied,^3,6–8^ a full genetic understanding of many emerging resistant clones, their role in exchanging genetic material, and the evolution of antimicrobial resistance (AMR) remains to be fully understood.

To date, a few studies have reported strains belonging to ST32 (Institute Pasteur scheme) in clinical and environmental samples.^9–13^ Notably, clinical ST32 isolates have been associated with resistance to many antibiotics, including carbapenems due to *bla*_OXA-58_ and aminoglycosides, and have been reported in different geographical areas including South Korea, Europe, the USA, Canada and the Middle East.^9–13^ However, these studies provide very limited amounts of genome sequence data, phylogenetic analyses or details about their population structure.

Here, we examined the genome sequence of 133 ST32 isolates, including 113 from the MRSN (the Multi-drug-Resistant organism repository and Surveillance Network). The latter were isolated in US military treatment facilities (MTFs) in the USA and Iraq between 2003 and 2004 and a single strain recovered in 2022 in a US MTF in Thailand. It has been shown that conflicts often results in increased incidence and transmission of multi-drug resistance microorganisms.^14,15^ Hence, examining historical *A. baumannii* genomes from the Middle East region is crucial in understanding the evolution of resistance, in particular in isolates linked to strains from this region. Here, we show that ST32 strains are present in diverse geographical areas including both natural environments and clinical samples. Our phylogenetic analysis shows that their population structure consists of several distinct clades, each specific to a region and exhibiting unique characteristics. We also provide evidence on the significant role of ST32 strains in the exchange of resistance determinants and evolution of AMR in several important STs including ST1 and ST3.

## Material and methods

### Antimicrobial susceptibility testing

Antibiotic susceptibility testing against 19 antibiotics (ampicillin/sulbactam, ampicillin, aztreonam, cefazolin, cefepime, ceftazidime, ceftriaxone, imipenem, meropenem, ciprofloxacin, levofloxacin, moxifloxacin, tetracycline, tigecycline, nitrofurantoin, tobramycin, gentamicin, amikacin and trimethoprim–sulfamethoxazole) was performed in a College of American Pathologists-accredited lab using a Vitek 2 with card GN AST 71.

### Genome sequence data

One hundred and thirty-three (*n *= 133) ST32 genomes were studied here. This included 113 genomes that belong to the MRSN project and were provided for this study. The MRSN strains were cultured from injured service members receiving treatment at MTFs in the USA or Germany in 2003–2004. The additional 20 ST32 genomes were found in GenBank non-redundant and draft genome databases and were included in this study. These 20 genomes were found by determining the ST and screening all publicly available *A. baumannii* genome sequence data. Meta-data and general properties of all strains are included (Table 1).

**Table 1.** General properties of *A. baumannii* strains containing Tn*6168*

Strain	Date	Source	Country	ST	Tn*6168* location	Accession #
J9	1999	Human	Australia	49	Plasmid	CP041590
AB0057	2004	Blood	USA	1	RS00030-035^a^	CP001182
A85	2003	Sputum	Australia	1	RS00030-035	CP021782
USA15	2013	Sputum	South Korea	1	RS00030-035	CP020595
Canada BC-5	2007	Hospital	Canada	1	RS00030-035	CP116680
NCSR_106	2007	Human	Vietnam	1	RS00030-035	CP154372
NCTC13421	2004	Human	UK	1	RS00030-035	LS483472
G20AB22	2020	Human	South Korea	1	RS00030-035	CP146812
C20AB12	2020	Human	South Korea	1	RS00030-035	CP142667
FDAARGOS_1306	NA	NA	USA	1	RS00030-035	CP066016
C20AB05	2020	Human	South Korea	1	RS00030-035	CP143262
ATCC BAA1605	2006	Sputum	Canada	1	RS00030-035	CP058625
AB6870155	2002	Sputum	Australia	1	RS00030-035	CP114381
CI300	2015	Tracheal aspirate	Lebanon	85	RS12940-945^b^	CP082952
B84	2017	Human	Germany	85	RS12940-945	CP139836
AB186-VUB	2017	Human	Belgium	85	RS12940-945	CP091356
AB177-VUB	NA	Human	Belgium	85	RS12940-945	CP091361
ACN21	2018	Blood	India	85	RS12940-945	CP038644
RAB57	2019	Human	Saudi Arabia	164	RS17670-675^c^	CP121570
DETAB-R21	2021	Rectal swab	China	164	RS17670-675	CP088895
JUNP496	2019	Human	Nepal	164	RS17670-675	AP031581
SRM3	2022	Human	China	164	RS17670-675	CP144242
XH1935	2021	Sputum	China	164	RS17670-675	CP088894

NA, not available.

^a^Tn*6168* located between ABA1_RS00030 and ABA1_RS00035. Locus IDs are based on the A1 genome (GenBank accession number CP010781).

^b^Tn*6168* located between ABA1_RS12940 and ABA1_RS12945.

^c^Tn*6168* located between ABA1_RS17670 and ABA1_RS17675.

### Whole genome sequencing, genome assembly and sequence analysis

For all 113 MRSN isolates, WGS was performed using an Illumina MiSeq benchtop sequencer, as previously described.^16^ Short-read sequencing data were trimmed for adapter sequence content and quality using Btrim64.^17^ Overlapping sequence reads were merged using FLASH.^18^  *De novo* assembly was performed using Newbler (v2.7) (https://github.com/etheleon/newbler). Minimum thresholds for contig size and coverage were set at 200 bp and ×4.95, respectively.

MGEs were identified using *ISFinder*^19^ and Standalone *BLAST.*^20^ Antibiotic resistance genes were identified using the *ABRicate*^21^ software, which uses the *ResFinder*^22^ and *CARD*^23^ databases. Protein coding regions were characterized using *BLASTp*^24^ and *UniProt*^25^ searches. STs were determined using the Institute Pasteur and Oxford MLST schemes using the *mlst* programme.^26^ The capsular polysaccharide (KL) and lipo-oligosaccharide outer core (OCL) synthesis loci were identified using *Kaptive*.^27^ Plasmid Rep types (*rep* gene types encoding replication initiation genes) were identified using the *Acinetobacter* plasmid typing scheme.^28,29^ The *SnapGene*^®^ v6.0.5 software was used to manually annotate regions of interest and draw figures to scale using the *Illustrator^®^* v26.2.1 programme.

### Phylogenetic analysis

The phylogenetic trees were constructed using Snippy-aligned sequences and filtered for recombination using *Gubbins* v.2.4.1. High-quality core-genome SNPs identified by *Gubbins*, which differentiated the genomes, were extracted to construct phylogenetic trees. Final maximum likelihood phylogenetic trees were inferred from the resulting alignment using *RAxML* (v.8) with the generalized time-reversible gamma model of nucleotide substitution, as previously described. Trees were visualized using Figtree.^30^ AMR genes and the phylogenetic tree were visualized using the *ggtree*,^31^  *ggplot2*^32^ and *plotTree*^33^ packages in R.

## Supplementary Material

dkae454_Supplementary_Data

## Figures and Tables

**Figure 1.**
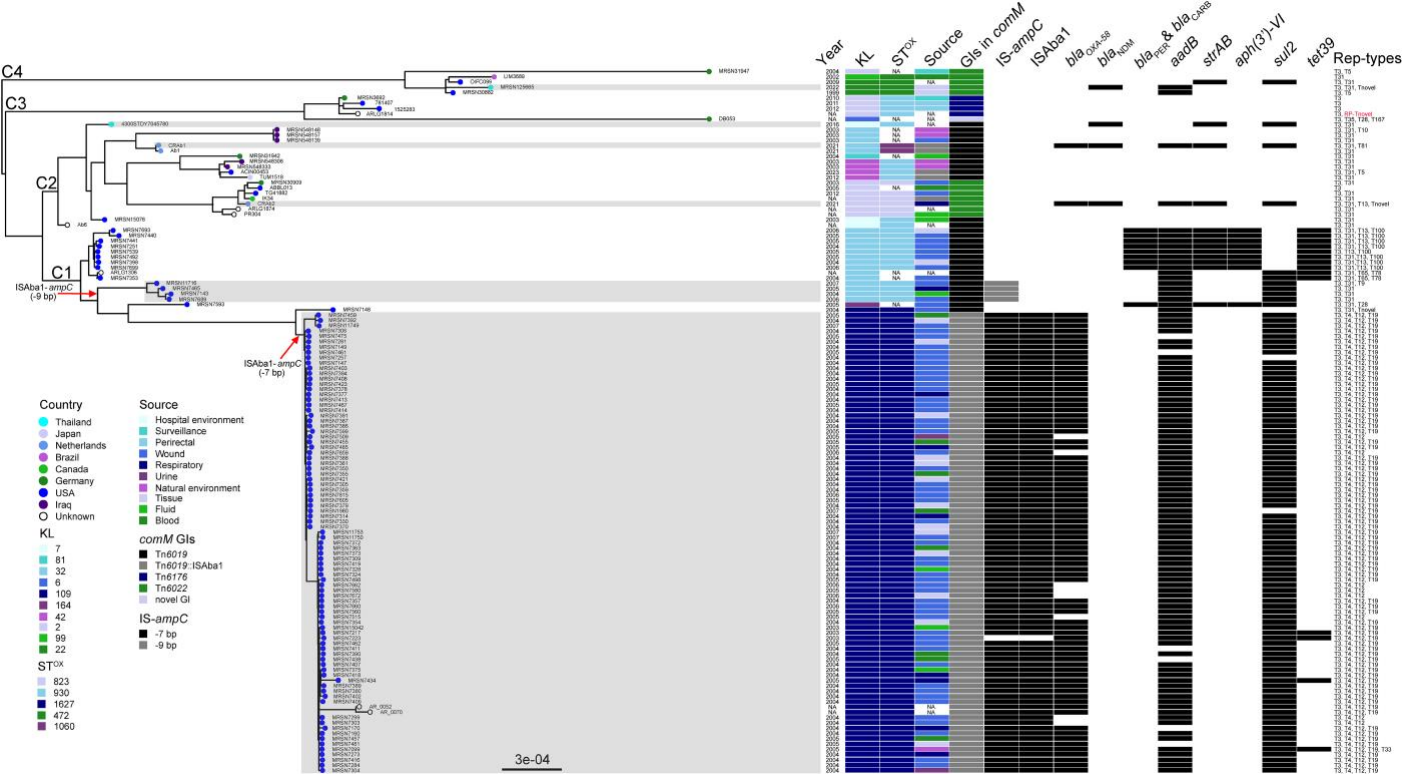
A phylogenetic tree depicting the genetic relationship between ST32 isolates. Colours in the tree’s node tips represent the country of origin of each strain, as depicted in the figure legend. Red arrows pointing to specific branches on the tree indicate acquisition points in clonal ancestry where successive strains share a common genetic component. Relevant metadata is plotted to the right of the phylogenetic tree, where respective data are aligned with the tip points of each strain. Plots labelled with ‘NA’ represent data that was not publicly available. The column headings ‘KL’ and ‘ST^OX^’ are indicative of the strain’s capsular polysaccharide K locus and Oxford scheme multi-locus ST, respectively. ‘GIs in *comM*’ refers to the presence of acquired GIs interrupting the intrinsic chromosomal *comM* gene. ‘IS-*ampC*’ represents the presence of ISAba1 directly upstream the start codon of *ampC*, while ‘ISAba1’ indicates the presence of ISAba1 closely downstream to *ampC*, to form a Tn*6168*-like structure. Relevant variations of each column heading are represented as different colours and are shown in the figure legend. C1–4 refers to Clades 1–4. The Rep types listed in the figure correspond to plasmid replication genes, with all but one (highlighted in red) belonging to the R3-type.

**Figure 2.**
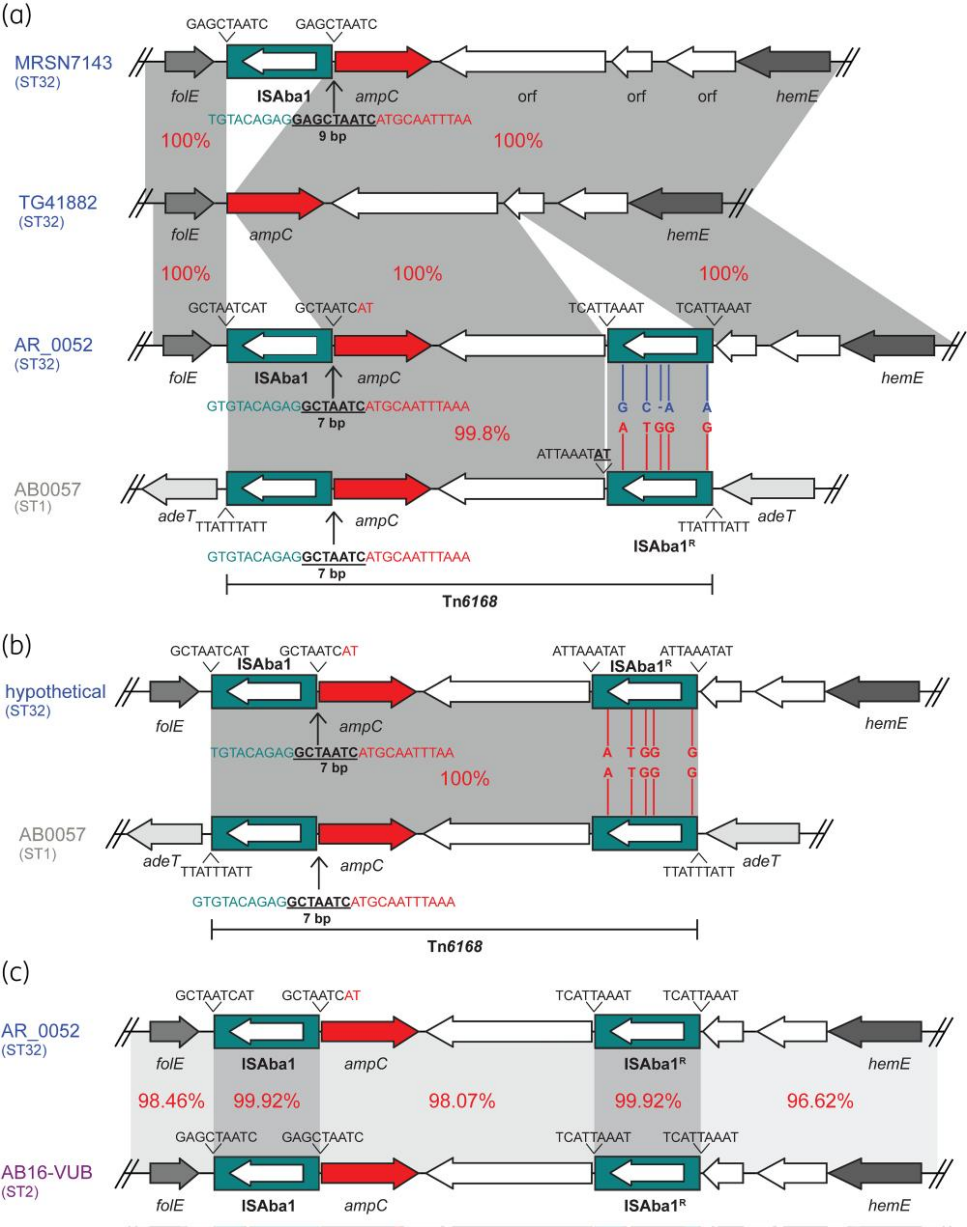
Schematic representation of the *ampC* region in ST32s and predicted origin and ancestral formation of Tn*6168*. Horizontal arrows represent the length and orientation of genes with *ampC* coloured red and insertion sequences represented as a green box with an internal white arrow to indicate the transposase. Strain names have been colour coded based on their respective Pasteur-scheme multi-locus ST. Parallel slanted lines demonstrate that the strain’s sequence goes beyond the region that is depicted within the figure. The 9 bp sequences shown above and below the flanking regions of insertion sequences indicate the target site duplication of each genetically incorporated element. Regions with significant DNA identities are highlighted in shades of grey, with percentage identities indicated by red numbers. (a) Shows the comparison between publicly available ST32 genomes and an ST1 genome containing Tn*6168*. (b) Shows a hypothetical ST32 genome that that has given rise to Tn*6168*. (c) Shows the comparison of ISAba1 insertion points around the *ampC* region between ST32 and ST2 strains (AB16-VUB also represent the second FDAARGOS_1359; the second ST2 with identical sequence). In panel (a) and (b) the tri-coloured sequence (shown in green, black and red) underneath specific strains outline the sequence around the start codon of the *ampC* gene, highlighting the variation in insertion points of ISAba1 upstream of *ampC*. The arrow directly above the sequence points to the location in the chromosome where this sequence is relevant. Further, in (a) red and blue lines protruding from ISAba1 highlight the location of SNPs between ST32 and Tn*6168*, while in (b) the uniform red sequence indicates and ISAba1 with no variation between the hypothetical sequence and Tn*6168*. ISAba1^R^ indicates the ISAba1 on the right-hand side.

**Figure 3.**
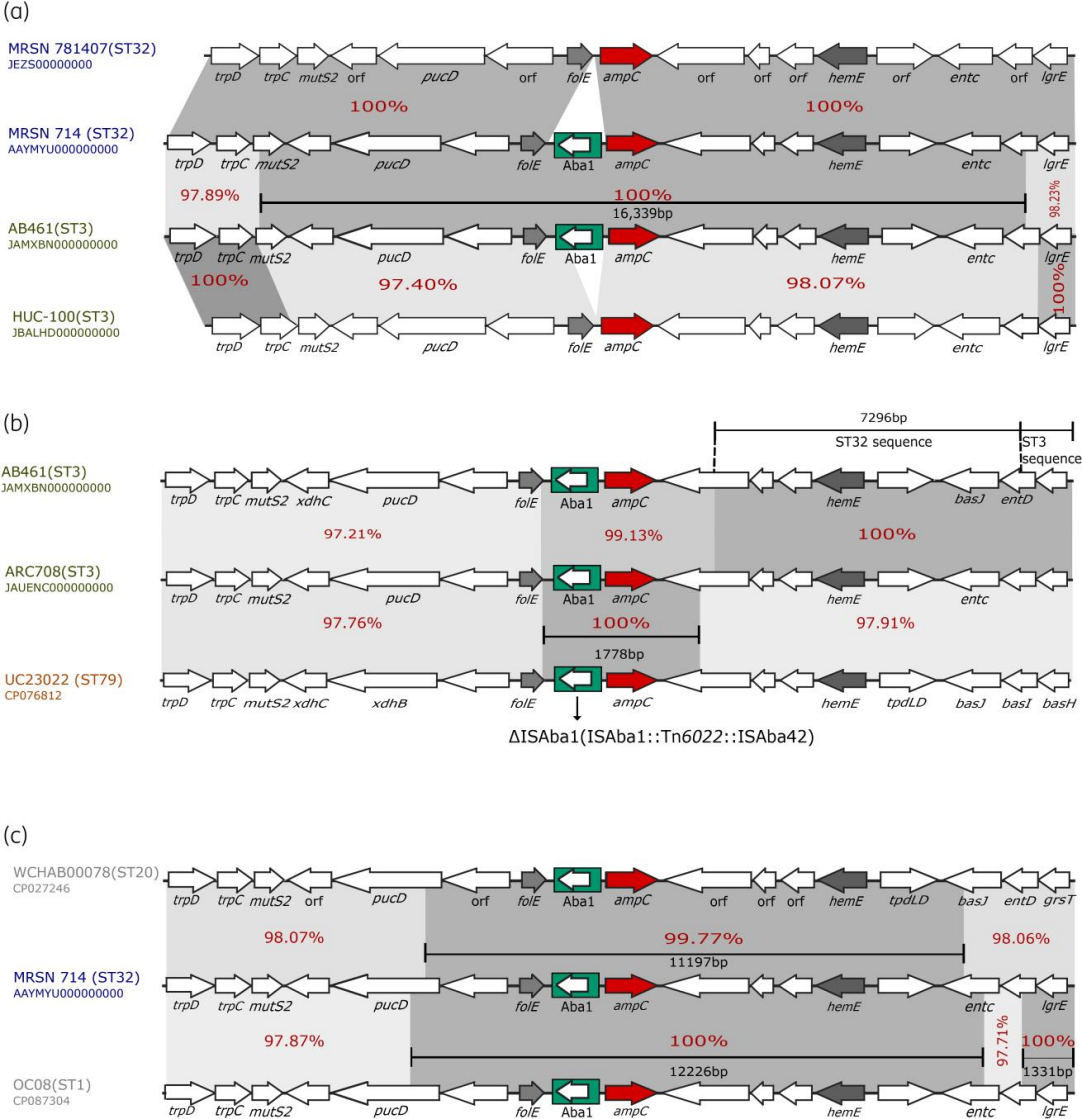
Schematic representation of the *ampC* chromosomal segments acquired by various STs. Horizontal arrows indicate the extent and orientation of genes, with the thin horizontal lines representing the boundaries for the segments acquired through homologous recombination. The red arrows represent the *ampC* gene, while the green boxes represent insertion sequences. Regions with significant DNA identities are highlighted in using shades of grey, with percentage identities indicated by red numbers. (a) Shows the recombinant *ampC* region in ST3 genomes with a 16.3 kb region acquired from ST32s, (b) shows a set of ST3s with a remnant of the ST32 recombinant region plus an additional segment acquired from ST79 strains and (c) shows the comparison of the *ampC* region in GC1 strains with an ISAba1 copy interrupting the downstream region of orf—like that in ST32s.

**Figure 4.**
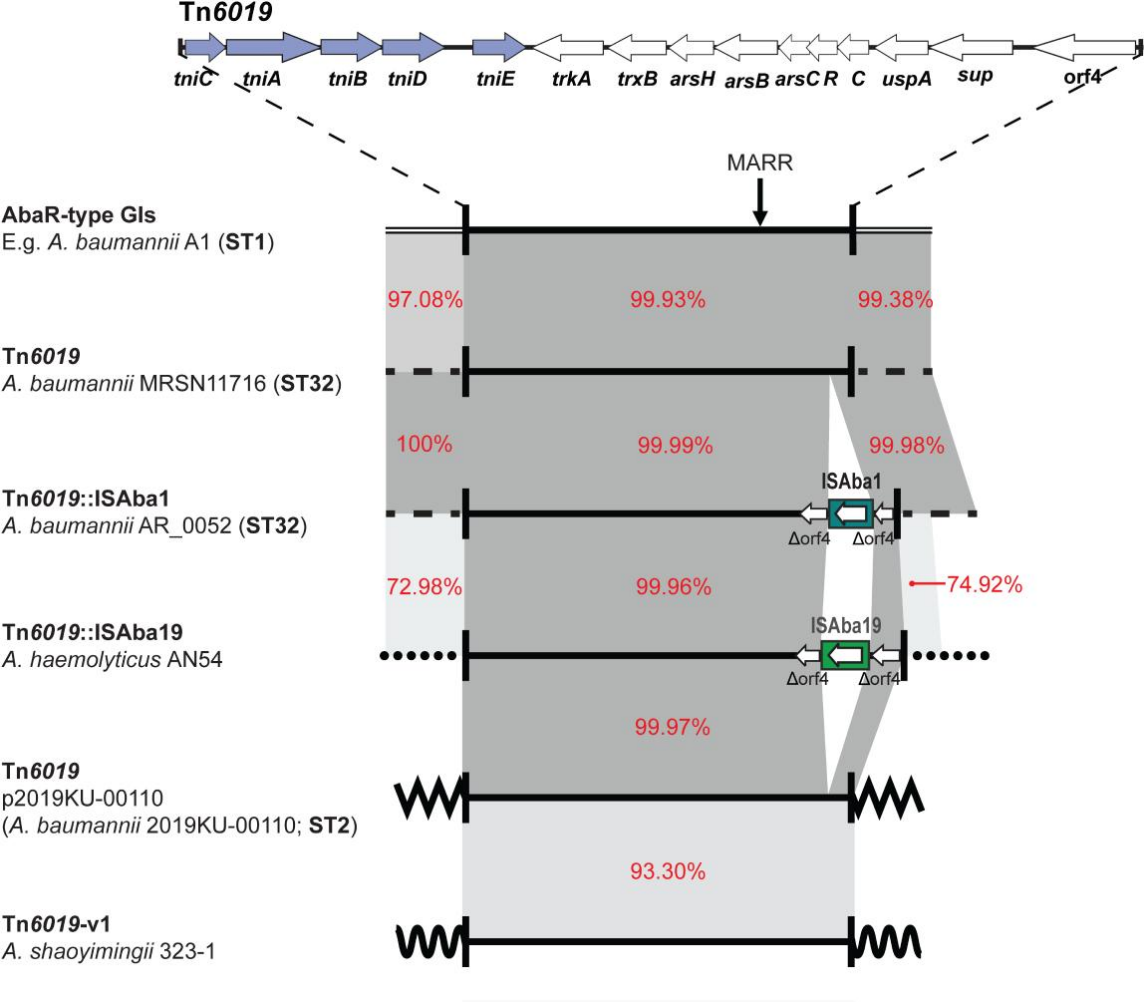
A schematic of the genetic context in which Tn*6019* and its variants were found in a diverse set of *Acinetobacter* strains. The arrows in the Tn*6019* structure indicate the boundaries and orientation of genes that make up Tn*6019*. Insertion sequences are represented by green boxes with an internal white arrow to represent their transposase. Regions with significant DNA identities are highlighted in shades of grey, with percentage identities indicated by numbers. MARR indicates the presence of a MARR, and its respective arrow indicates its location with Tn*6019* in AbaR-type GIs. The varying patterned lines beyond the Tn*6019* structure highlights the variation in genetic backbone (different STs and/or species) and shows the difference in genetic context in which Tn*6019* is found.

**Table 2.** Properties of the *Acinetobacter* genomes carrying Tn*6019* and its variants

Species	Strain	Date	Country	Source	ST^IP^	Tn*6019* (and variants)	Tn*6019* location	GenBank no.
*A. baumannii*	NCTC13305	NA	UK	NA	–	Tn*6019*	*comM* ^a^	UFSC01000003
*A. baumannii*	Ab1	NA	UK	NA	–	Tn*6019*	*comM*	CAMZOM010000000
*A. baumannii*	Ab6	NA	UK	NA	–	Tn*6019*	*comM*	CAMRHY010000000
*A. baumannii*	Whole organism	2019	USA	Groin	734	Tn*6019*	Novel R3-type plasmid	ABDUQV030000016
*A. baumannii*	2019KU-00110	2019	USA	NA	2	Tn*6019*	RP-T1 plasmid	ABDOFH030000008
*A. lwoffii*	ERR9969097_bin.4^b^	2014	Australia	Oral metagenome	–	Tn*6019*	*comM*	CAUQLC010000000
*A. lwoffii*	WU_MDCI_Al101	2018	USA	Respiratory infection	–	Tn*6019*	*comM*	JAHPSO010000000
*A. haemolyticus*	AN54	2016	Mexico	Peritoneal fluid	1934	Tn*6019*::ISAba19	*comM*	CP041224
*A. shaoyimingii*	323-1	2017	China	Animal (Equus kiang)	1798	Tn*6019-v1*	*comM*	CP049801
*A. radioresistens*	C21A	2012	Panama	Skin	–	Tn*6019*	*comM*	JAMOZF010000000
*A. radioresistens*	C30P	2012	Panama	Skin	—	Tn*6019*	*comM*	JAMOZE010000000

NA, not available.

^a^Tn*6019* located in the *comM* gene in the chromosome.

^b^Abbreviated here; complete strain name is ERR9969097_bin.4_MetaWRAP_v1.3_MAG.

## Data Availability

All MRSN ST32 strains have been deposited in the GenBank/EMBL/DDBJ databases and are publicly available under the following BioProjects: PRJNA1134881, PRJNA300270, PRJNA545079, PRJNA300270, PRJNA53379, PRJNA53459 and PRJNA53461. Specific GenBank accession numbers for all genomes studied here are also included in Table S1 (available as Supplementary data at *JAC* Online).

## Results and discussion

### General properties and phylogenetic relationship

Here, we studied the evolution of antibiotic resistance and phylogenomics of a total of 133 ST32 genomes, the majority of which (*n *= 113) were recovered from both clinical samples and hospital environments in US MTFs in the Middle East (mainly Fallujah, Iraq), the USA or Germany. Although the majority (*n *= 113) were cultured from US Service Members injured in the Middle East, 20 strains were also from other global regions including Thailand, Canada and Brazil, indicative of their broader geographical distribution (Figure 1).

Analysis of the surface polysaccharides indicated all strains encode the OCL6 lipo-oligosaccharide outer core. Although most strains (*n *= 89) contained the KL109 capsular polysaccharide (CPS) locus (Figure 1), nine (*n *= 9) other KL types were also identified. All KL109 strains belonged to ST1627 (Oxford scheme). Consistent with KL variations, all strains with non-KL109 also belonged to different Oxford scheme STs (Figure 1) due to the inclusion of the *gpi* gene, which is part of the K locus in this Oxford MLST scheme.^34,35^ This CPS variability in our strains is consistent with previous studies that have noted the highly variable nature, with even closely related isolates producing different forms of CPS.^34,35^

The phylogenetic tree of the core genomes comprises four (*n *= 4) main clades (named C1–4 in Figure 1), each containing several sub-clades. Although MRSN genomes were dispersed across C1–4, the majority (*n *= 103) fell within the C1 clade, with over 80 strains forming a tight cluster, indicating their close relationship. This clustering suggests they are likely to represent outbreak strains (hence hereafter referred to as outbreak strains). Core-genome SNP analysis revealed that these strains differed from each other by only 10–50 SNPs but were separated from all other strains by 100 to >1200 SNPs (Table S1).

### Antibiotic resistance profiles and distribution of resistance genes

All strains were susceptible to amikacin (Table S2). Eighty-eight strains (*n* = 88) exhibited resistance to tobramycin, kanamycin and gentamicin, likely due to the presence of the *aadB* gene on the small 6 kb plasmid pRAY (Figure 1). The pRAY plasmid has been shown to play an important role in the dissemination of the *aadB* gene across various *Acinetobacter* species, *A. baumannii* STs and geographic regions.^36^ Amongst these strains with *aadB*, eight (*n* = 8) belonging to two distinct lineages also carried the *sul2* sulfonamide resistance gene, along with the beta-lactam resistance genes *bla*_CARB_ and *bla*_PER_, and the aminoglycoside resistance genes *strAB* and *aph(3*′*)-VI* (Figure 1). Sequence analysis predicted that they are likely carried on a <20 kb plasmid encoding two replication proteins (Reps), R3-T13 and R3-T100. Specifically, the *bla*_PER_ ESBL resistance gene was found within the known ISPa12 and ISPa13 bounded transposon Tn*1213*.^37^

No IS was found upstream of the intrinsic *oxaAb* (also known as *bla*_OXA-51-like_) gene in all ST32s studied here. However, all outbreak strains contained a copy of the *bla*_OXA-58_ Class D carbapenem resistance gene. The gene was flanked by an ISAba3 and a ΔISAba3 in a *dif* module identical to that we recently described in the pA388 plasmid (GenBank accession number CP024419).^38,39^ Analysis of the contigs with *bla*_OXA-58_ and correlation with the presence of an R3-T19 suggests that an R3-type plasmid that encodes R3-T19 carries the *bla*_OXA-58_ gene, but long-read sequencing would be required to confirm this.

Four strains (MRSN125665 and 4300STDY7045780, recovered in Thailand, and Crab1 and Crab2, recovered in the Netherlands; Figure 1) contained the *bla*_NDM_ carbapenem-resistance gene. In all four genomes, the *bla*_NDM_ gene was in Tn*125*, a known composite ISAba125-bounded transposon. Tn*125* is present in a novel plasmid in the two Dutch strains (Crab1 and Crab2). However, its context could not be resolved in the Thai strains due to the presence of at least 19 ISAba125 copies, which resulted in several (*n *= 39) broken contigs. Unlike in Enterobacterales, *bla*_NDM_ is not a common carbapenem resistance gene in *A. baumannii*.^1^ Therefore, it is significant that these ST32 strains from unrelated regions carry this important carbapenem resistance gene.

### Evolution of the *ampC* region and links to origin of Tn*6168*

ISAba1 often inserts 9 bp upstream of the ATG (start codon) of the *ampC* gene and is responsible for enhancing the expression of *ampC*, leading to resistance to third-generation cephalosporins (e.g. ceftazidime and cefotaxime).^40^ Analysis of this *ampC* region in ST32 genomes indicated that 91 genomes include a copy of ISAba1 upstream of this gene. Forty-two (*n *= 42) strains did not include any IS in the region surrounding *ampC*, indicating that they contain an intact structure in this chromosomal region (Figure 1, and TG41882 in Figure 2a). Consistent with the presence of ISAba1, all strains with an IS-*ampC* showed either resistance or reduced susceptibility to third-generation cephalosporins (Table S1).

Amongst the genomes with an ISAba1-*ampC* structure (*n *= 91), in four (*n *= 4) genomes (MRSN11716, MRSN7465, MRSN7143 and MRSN7689; Figure 2a) the ISAba1 was 9 bp away from the ATG of *ampC*, which is precisely where the IS is often found in this chromosomal location.^40^ These four strains formed a small phylogenetic branch, indicating they are closely related and that the IS has been acquired by their ancestral strain of this small branch (marked with a red arrow and as −9 bp ISAba1-*ampC* in Figure 1). Analysis of the remaining genomes with an ISAba1 upstream of *ampC* (*n *= 87) indicated that the IS is present 7 bp away from the start of *ampC*. This is reminiscent of Tn*6168*,^41^ as this transposon is the only known structure to include the IS 7 bp from the ATG of *ampC*. We previously reported that the *ampC* sequence in Tn*6168* is virtually identical to a few ST32 and ST3 genomes that were available at the time.^41^ Therefore, to better understand the origin of this important transposon, we further analysed this region in ST32 and ST3 strains, which contained similar *ampC* sequences. In all ST32 strains, the sequence of the *ampC*-orf region differs by only 1–3 bp compared to the corresponding region in Tn*6168*. In addition, in all outbreak strains (with IS 7 bp away from the *ampC* ATG), there was a second ISAba1 copy (called ISAba1^R^ hereafter for simplicity) 2 bp away from where it would be in Tn*6168* (Figure 2a and b). It is tempting to consider this version (with two ISAba1 copies) as the source for Tn*6168*. However, the central fragment of Tn*6168* is 2 bp shorter than the corresponding region in ST32s (the region between two ISAba1 copies), with the 2 bp being immediately adjacent to ISAba1^R^. It is possible that the 2 bp sequence (AT, indicated in bold and underlined in Figure 2a) has been deleted via an ISAba1 adjacent deletion leading to the formation of Tn*6168*. However, it is also likely that Tn*6168* originated from another intermediate ST32 that includes the second IS exactly where it is in Tn*6168* (and 2 bp away from the ISAba1^R^ position in AR_0052 and all other ST32 genomes with the same structure; Figure 2). The latter is further supported by the fact that the sequence of ISAba1^R^ (flanked by the TCATTAAAT target site duplication; Figure 2a and b) differs from the corresponding IS copy in Tn*6168* by 5 SNPs (i.e. GC-AA versus ATGGG, Figure 2a). Hence, we propose that Tn*6168* was formed by the excision of this Tn from an ancestral ST32 with an ISAba1^R^ identical to that in Tn*6168* (Hypothetical strain in Figure 2b).

Interestingly, we also found two ST2 strains (AB16-VUB; GenBank accession number CP091375.1 and FDAARGOS_1359; GenBank accession number CP069851.1), with an ISAba1 copy 9 bp upstream of the *ampC* start codon. These ST2s also contained an ISAba1^R^ copy precisely where ISAba1^R^ is present in all ST32s with two IS flanking the *ampC* region (Figure 2c). The presence of the IS 9 bp upstream and just 98% *ampC*-orf sequence identity (DNA sequence) makes these structures unrelated to ST32 strains. However, it raises the question of why the second IS has inserted precisely in the same chromosomal location in two unrelated clones with no obvious benefit (i.e. no gene to activate). This remains to be established.

Additionally, given the significance of Tn*6168* in the spread of an activated *ampC* third-generation cephalosporin resistance determinant, we explored all complete genomes to track its movement. In addition to the ST1^2^ strains and pJ9-3 we previously reported,^42^ Tn*6168* was found in several strains belonging to ST85 and ST164 from different geographical areas, indicating its wide spread (Table 1). This increases the significance of Tn*6168* given that it has mobilized to several important globally distributed MDR clones (e.g. ST1, ST85 and ST164). In each ST, Tn*6168* was in a different chromosomal location, indicating that Tn*6168* has been acquired by each ST via a distinct transposition event (Table 1).

### Several ST3 strains acquired an ISAba1-*ampC* from ST32s via homologous recombination

We have previously shown that the *ampC* gene can be activated in multiple ways including the upregulation by ISAba1, the acquisition of Tn*6168* or the acquisition of a chromosomal segment with an IS-activated *ampC* through homologous recombination.^2,40,41,43^

To examine the relationships of the *ampC* region in ST32s, Tn*6168* and ST3 strains, we performed a comparative sequence analysis of this region in all publicly available ST3 genomes (*n *= 144 genomes; last accessed April 2024). Sequence analysis of 20 kb downstream and upstream of *ampC* showed that an ISAba1-activated *ampC* was acquired as a 16 409 bp segment in *n *= 127 ST3 genomes via homologous recombination (e.g. strain AB461; GenBank accession number JAMXBN000000000; Figure 3a). The sequence of this ∼16.5 kb region was 100% identical to the corresponding sequence in ST32s. The flanking regions of this recombinant sequence were 2%–3% different compared to ST32s and 100% identical compared to 10 ST3 genomes, which included the original ST3 sequence that lacks ISAba1 (e.g. strain HUC-100; GenBank accession number JBALHD000000000; Figure 3a). HUC-100 and nine additional genomes contained the original ST3 sequence as we did not detect any recombination signals in their *ampC* regions.

Seven ST3 genomes, represented by strain ARC708 (GenBank accession number JAUENC000000000 in Figure 3b), retained a partial (7.3 kb) fragment of the ST32 recombinant segment but also included a second 2511 bp segment, which differed by at least 2% DNA identity compared to both ST32 and ST3 genomes. Sequence analysis to find the source of this region showed that it has been imported from ST79 strains (such as strain UC2302 shown in Figure 3b; GenBank accession number CP076812).

Furthermore, we also found two genomes that belong to the global clone 1 clonal complex (WCHAB00078 belonging to ST20; GenBank accession number CP027245 and OC08 belonging to ST1; GenBank accession number CP087304) in which their *ampC* sequences were 100% identical to that in ST32s. We found two recombinant DNA segments of 7195 and 12 226 bp, respectively, in both strains (Figure 3c) with 100% DNA identity compared to ST32s, indicating acquisition via recombination.

Together, the analysis of the *ampC* region indicated that ST32s appear to have played a significant role in supplying activated *ampC* genes to several strains that belong to important STs including ST1 and ST3.

### Multiple ST32 lineages carry Tn*6019* in the chromosomal *comM* gene

In most members of the globally distributed ST1 isolates, the chromosomal *comM* gene is often interrupted with a variant of the AbaR-type genomic resistance islands, which contain a transposon backbone called Tn*6019*.^44^ To identify GIs and/or transposons inserted in the *comM* gene, we analysed this gene in all ST32s. The *comM* gene was found to be interrupted by an insertion in every genome. In four genomes (ARLG, 1525283, 78407 and MRSN3692), clustered in a small branch in the tree, the *comM* gene was disrupted by Tn*6176*. Tn*6176* is a known copper GI (Figure 1) with a backbone related to the Tn*6019* and Tn*6022* family.^42,45^ Ten (*n *= 10) strains had an intact copy of Tn*6022* inserted in *comM*. Tn*6022* is the backbone of AbaR4, which carries the *bla*_OXA-23_ carbapenem resistance gene and has been shown to target *comM*.^46^ These strains fall into two small branches and belong to diverse geographical areas (Figure 1).

The remaining strains contained either an intact copy of Tn*6019* or its ISAba1-interrupted version (Tn*6019*::ISAba1) within *comM*. Notably, those with Tn*6019*::ISAba1 belong to the tightly clustered outbreak US MTF isolates (Figure 1). The presence of Tn*6019* in the *comM* gene of several ST32s (excluding those genomes we are releasing to the public domain here) has also been noted on a recently developed *Acinetobacter* web page (https://acinetobacterbaumannii.no).

Given the significance of Tn*6019* in the structure of AbaR-type GIs, we tracked this transposon and found it in several *A. baumannii* and non-*A*. *baumannii* strains, both in the *comM* gene and in plasmid contexts (Table 2 and Figure 4). Notably, some of these strains were recovered in metagenomic or animal studies, indicating the presence of these elements in non-clinical settings—likely before they captured the multiply antibiotic resistance region (MARR) and turned into the AbaR-type genomic resistance islands as we previously hypothesized.^44^

### Conclusions

Most ST32s available are linked to the conflict in the Middle East. Close relatives of the Middle Eastern ST32 strains appear to have played a role in supplying third-generation cephalosporin determinants (ISAba1-activated *ampC*) to several significant STs via recombination. However, more ST32 strains from the Middle East and other geographically diverse regions would need to be sequenced and analysed to better understand the evolution of resistance in this clone and their exchange of genetic material with other clones. Tn*6168,* a composite transposon carrying an ISAba1-activated *ampC* gene, has also mobilized the *ampC* gene from an ST32 *A. baumannii* strain.

## Supplementary data

Table S1 is available in https://doi.org/10.6084/m9.figshare.26309839.

Table S2 is available via https://doi.org/10.6084/m9.figshare.26309824.
